# Supplementary figures and images for: Proteomic analysis of IgM antigens from mammary tissue under pre- and post-cancer conditions using the MMTV-PyVT mouse model
Source: PeerJ. 2022 Oct 18;10:e14175. doi: 10.7717/peerj.14175 (PMC9586126; doi:10.7717/peerj.14175)

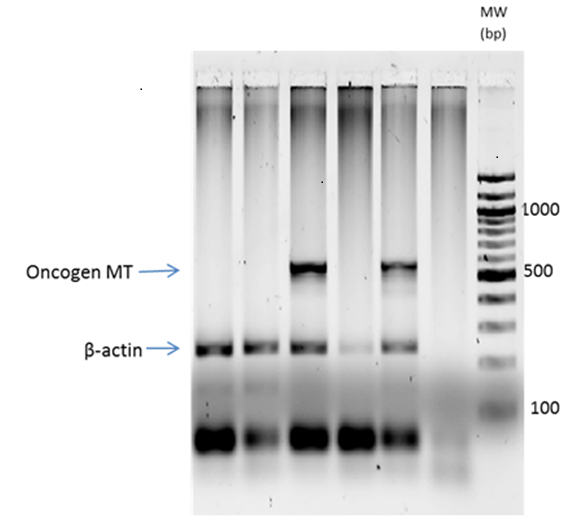

Supplement: Figure S1 — Identification by PCR of newborn female PyVT mice carrying the MT oncogene [file peerj-10-14175-s001.tif]

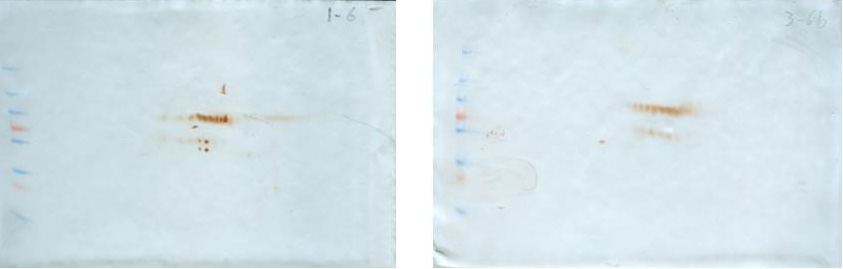

Supplement: Figure S2 — 2D immunoblot of antigenic recognition by serum IgM of normal female mice (A) and Transgenic mice (B) on the membrane fraction of mammary tissue at the sixth week of age. [file peerj-10-14175-s002.jpg]
