# Supplementary material for: Proteomic analysis of IgM antigens from mammary tissue under pre- and post-cancer conditions using the MMTV-PyVT mouse model
Source: PeerJ. 2022 Oct 18;10:e14175. doi: 10.7717/peerj.14175 (PMC9586126; doi:10.7717/peerj.14175)
Supplement: Supplemental Information 2 [file peerj-10-14175-s004.zip › Raw data/Legends.docx]

- AD_CHRO001.DAT, Raw data exported from the Synapt G2S Model Mass Spectrometer (WATERS), applied for data analyses and preparation for the sequence of spot A duplicate in table 1

- AD_FUNC002.IDX, Raw data exported from the Synapt G2S Model Mass Spectrometer (WATERS), applied for data analyses and preparation for the sequence of spot A duplicate in table 1

- AD_extern.inf, Raw data exported from the Synapt G2S Model Mass Spectrometer (WATERS), applied for data analyses and preparation for the sequence of spot A duplicate in table 1

- AD_FUNC001.IDX, Raw data exported from the Synapt G2S Model Mass Spectrometer (WATERS), applied for data analyses and preparation for the sequence of spot A duplicate in table 1

- AD_FUNC003.IDX, Raw data exported from the Synapt G2S Model Mass Spectrometer (WATERS), applied for data analyses and preparation for the sequence of spot A duplicate in table 1

- AD_FUNCTNS.INF, Raw data exported from the Synapt G2S Model Mass Spectrometer (WATERS), applied for data analyses and preparation for the sequence of spot A duplicate in table 1

- AD_FUNC002.STS, Raw data exported from the Synapt G2S Model Mass Spectrometer (WATERS), applied for data analyses and preparation for the sequence of spot A duplicate in table 1

- AD_INLET.INF, Raw data exported from the Synapt G2S Model Mass Spectrometer (WATERS), applied for data analyses and preparation for the sequence of spot A duplicate in table 1

- AD_HEADER.TXT, Raw data exported from the Synapt G2S Model Mass Spectrometer (WATERS), applied for data analyses and preparation for the sequence of spot A duplicate in table 1

- AD_FUNC001.STS, Raw data exported from the Synapt G2S Model Mass Spectrometer (WATERS), applied for data analyses and preparation for the sequence of spot A duplicate in table 1

- AD_FUNC003.STS, Raw data exported from the Synapt G2S Model Mass Spectrometer (WATERS), applied for data analyses and preparation for the sequence of spot A duplicate in table 1

- AD_CHROMS.INF, Raw data exported from the Synapt G2S Model Mass Spectrometer (WATERS), applied for data analyses and preparation for the sequence of spot A duplicate in table 1

- BD_FUNC001.IDX, Raw data exported from the Synapt G2S Model Mass Spectrometer (WATERS), applied for data analyses and preparation for the sequence of spot B duplicate in table 1

- BD_FUNC003.IDX, Raw data exported from the Synapt G2S Model Mass Spectrometer (WATERS), applied for data analyses and preparation for the sequence of spot B duplicate in table 1

- BD_FUNC002.IDX Raw data exported from the Synapt G2S Model Mass Spectrometer (WATERS), applied for data analyses and preparation for the sequence of spot B duplicate in table 1

- BD_extern.inf, Raw data exported from the Synapt G2S Model Mass Spectrometer (WATERS), applied for data analyses and preparation for the sequence of spot B duplicate in table 1

- BD_FUNC003.STS, Raw data exported from the Synapt G2S Model Mass Spectrometer (WATERS), applied for data analyses and preparation for the sequence of spot B duplicate in table 1

- BD_HEADER.TXT, Raw data exported from the Synapt G2S Model Mass Spectrometer (WATERS), applied for data analyses and preparation for the sequence of spot B duplicate in table 1

- BD_FUNC001.STS, Raw data exported from the Synapt G2S Model Mass Spectrometer (WATERS), applied for data analyses and preparation for the sequence of spot B duplicate in table 1

- BD_FUNC002.STS, Raw data exported from the Synapt G2S Model Mass Spectrometer (WATERS), applied for data analyses and preparation for the sequence of spot B duplicate in table 1

- BD_CHRO001.DAT, Raw data exported from the Synapt G2S Model Mass Spectrometer (WATERS), applied for data analyses and preparation for the sequence of spot B duplicate in table 1

- CD_CHRO001.DAT, Raw data exported from the Synapt G2S Model Mass Spectrometer (WATERS), applied for data analyses and preparation for the sequence of spot C duplicate in table 1

- CD_FUNC001.IDX, Raw data exported from the Synapt G2S Model Mass Spectrometer (WATERS), applied for data analyses and preparation for the sequence of spot C duplicate in table 1

- CD_FUNC001.STS, Raw data exported from the Synapt G2S Model Mass Spectrometer (WATERS), applied for data analyses and preparation for the sequence of spot C duplicate in table 1

- CD_HEADER.TXT, Raw data exported from the Synapt G2S Model Mass Spectrometer (WATERS), applied for data analyses and preparation for the sequence of spot C duplicate in table 1

- CD_FUNC002.STS, Raw data exported from the Synapt G2S Model Mass Spectrometer (WATERS), applied for data analyses and preparation for the sequence of spot C duplicate in table 1

- CD_extern.inf, Raw data exported from the Synapt G2S Model Mass Spectrometer (WATERS), applied for data analyses and preparation for the sequence of spot C duplicate in table 1

- CD_FUNC003.STS, Raw data exported from the Synapt G2S Model Mass Spectrometer (WATERS), applied for data analyses and preparation for the sequence of spot C duplicate in table 1

- CD_INLET.INF, Raw data exported from the Synapt G2S Model Mass Spectrometer (WATERS), applied for data analyses and preparation for the sequence of spot C duplicate in table 1

- CD_FUNC003.IDX, Raw data exported from the Synapt G2S Model Mass Spectrometer (WATERS), applied for data analyses and preparation for the sequence of spot C duplicate in table 1

- CD_FUNC002.IDX, Raw data exported from the Synapt G2S Model Mass Spectrometer (WATERS), applied for data analyses and preparation for the sequence of spot C duplicate in table 1

- DD_CHRO001.DAT Raw data exported from the Synapt G2S Model Mass Spectrometer (WATERS), applied for data analyses and preparation for the sequence of spot D duplicate in table 1

- DD_FUNC002.IDX, Raw data exported from the Synapt G2S Model Mass Spectrometer (WATERS), applied for data analyses and preparation for the sequence of spot D duplicate in table 1

- DD_FUNC001.IDX, Raw data exported from the Synapt G2S Model Mass Spectrometer (WATERS), applied for data analyses and preparation for the sequence of spot D duplicate in table 1

- DD_extern.inf, Raw data exported from the Synapt G2S Model Mass Spectrometer (WATERS), applied for data analyses and preparation for the sequence of spot D duplicate in table 1

- DD_FUNC001.STS, Raw data exported from the Synapt G2S Model Mass Spectrometer (WATERS), applied for data analyses and preparation for the sequence of spot D duplicate in table 1

- DD_FUNC003.STS, Raw data exported from the Synapt G2S Model Mass Spectrometer (WATERS), applied for data analyses and preparation for the sequence of spot D duplicate in table 1

- DD_HEADER.TXT, Raw data exported from the Synapt G2S Model Mass Spectrometer (WATERS), applied for data analyses and preparation for the sequence of spot D duplicate in table 1

- DD_FUNC002.STS, Raw data exported from the Synapt G2S Model Mass Spectrometer (WATERS), applied for data analyses and preparation for the sequence of spot D duplicate in table 1

- DD_FUNC003.IDX, Raw data exported from the Synapt G2S Model Mass Spectrometer (WATERS), applied for data analyses and preparation for the sequence of spot D duplicate in table 1

- A_CHRO001.DAT, Raw data exported from the Synapt G2S Model Mass Spectrometer (WATERS), applied for data analyses and preparation for the sequence of spot A in table 1

- A_extern.inf, Raw data exported from the Synapt G2S Model Mass Spectrometer (WATERS), applied for data analyses and preparation for the sequence of spot A in table 1

- A_FUNC003.IDX, Raw data exported from the Synapt G2S Model Mass Spectrometer (WATERS), applied for data analyses and preparation for the sequence of spot A in table 1

- A_FUNC001.STS, Raw data exported from the Synapt G2S Model Mass Spectrometer (WATERS), applied for data analyses and preparation for the sequence of spot A in table 1

- A_FUNC003.STS, Raw data exported from the Synapt G2S Model Mass Spectrometer (WATERS), applied for data analyses and preparation for the sequence of spot A in table 1

- A_FUNC001.IDX, Raw data exported from the Synapt G2S Model Mass Spectrometer (WATERS), applied for data analyses and preparation for the sequence of spot A in table 1

- A_FUNC002.STS, Raw data exported from the Synapt G2S Model Mass Spectrometer (WATERS), applied for data analyses and preparation for the sequence of spot A in table 1

- A_INLET.INF, Raw data exported from the Synapt G2S Model Mass Spectrometer (WATERS), applied for data analyses and preparation for the sequence of spot A in table 1

- A_FUNC002.IDX Raw data exported from the Synapt G2S Model Mass Spectrometer (WATERS), applied for data analyses and preparation for the sequence of spot A in table 1

- A_HEADER.TXT, Raw data exported from the Synapt G2S Model Mass Spectrometer (WATERS), applied for data analyses and preparation for the sequence of spot A in table 1

- B_CHRO001.DAT, Raw data exported from the Synapt G2S Model Mass Spectrometer (WATERS), applied for data analyses and preparation for the sequence of spot B in table 1

- B_FUNC002.IDX, Raw data exported from the Synapt G2S Model Mass Spectrometer (WATERS), applied for data analyses and preparation for the sequence of spot B in table 1

- B_FUNC003.IDX Raw data exported from the Synapt G2S Model Mass Spectrometer (WATERS), applied for data analyses and preparation for the sequence of spot B in table 1

- B_FUNC002.STS, Raw data exported from the Synapt G2S Model Mass Spectrometer (WATERS), applied for data analyses and preparation for the sequence of spot B in table 1

- B_extern.inf, Spot B, Raw data exported from the Synapt G2S Model Mass Spectrometer (WATERS), applied for data analyses and preparation for the sequence of spot B in table 1

- B_FUNC001.IDX Raw data exported from the Synapt G2S Model Mass Spectrometer (WATERS), applied for data analyses and preparation for the sequence of spot B in table 1

- B_HEADER.TXT, Raw data exported from the Synapt G2S Model Mass Spectrometer (WATERS), applied for data analyses and preparation for the sequence of spot B in table 1

- B_FUNC001.STS, Raw data exported from the Synapt G2S Model Mass Spectrometer (WATERS), applied for data analyses and preparation for the sequence of spot B in table 1

- B_FUNC003.STS, Raw data exported from the Synapt G2S Model Mass Spectrometer (WATERS), applied for data analyses and preparation for the sequence of spot B in table 1

- C_CHRO001.DAT, Raw data exported from the Synapt G2S Model Mass Spectrometer (WATERS), applied for data analyses and preparation for the sequence of spot C in table 1

- C_HEADER.TXT Raw data exported from the Synapt G2S Model Mass Spectrometer (WATERS), applied for data analyses and preparation for the sequence of spot C in table 1

- C_FUNC001.STS, Raw data exported from the Synapt G2S Model Mass Spectrometer (WATERS), applied for data analyses and preparation for the sequence of spot C in table 1

- C_extern.inf, Raw data exported from the Synapt G2S Model Mass Spectrometer (WATERS), applied for data analyses and preparation for the sequence of spot C in table 1

- C_FUNC003.STS, Raw data exported from the Synapt G2S Model Mass Spectrometer (WATERS), applied for data analyses and preparation for the sequence of spot C in table 1

- C_FUNC002.IDX, Raw data exported from the Synapt G2S Model Mass Spectrometer (WATERS), applied for data analyses and preparation for the sequence of spot C in table 1

- C_FUNC003.IDX, Raw data exported from the Synapt G2S Model Mass Spectrometer (WATERS), applied for data analyses and preparation for the sequence of spot C in table 1

- C_FUNC002.STS, Raw data exported from the Synapt G2S Model Mass Spectrometer (WATERS), applied for data analyses and preparation for the sequence of spot C in table 1

- C_FUNC001.IDX, Raw data exported from the Synapt G2S Model Mass Spectrometer (WATERS), applied for data analyses and preparation for the sequence of spot C in table 1

- D_CHRO001.DAT, Raw data exported from the Synapt G2S Model Mass Spectrometer (WATERS), applied for data analyses and preparation for the sequence of spot D in table 1

- D_extern.inf, Raw data exported from the Synapt G2S Model Mass Spectrometer (WATERS), applied for data analyses and preparation for the sequence of spot D in table 1

- D_FUNC003.IDX, Raw data exported from the Synapt G2S Model Mass Spectrometer (WATERS), applied for data analyses and preparation for the sequence of spot D in table 1

- D_FUNC001.STS, Raw data exported from the Synapt G2S Model Mass Spectrometer (WATERS), applied for data analyses and preparation for the sequence of spot D in table 1

- D_FUNC002.STS, Raw data exported from the Synapt G2S Model Mass Spectrometer (WATERS), applied for data analyses and preparation for the sequence of spot D in table 1

- D_FUNC002.IDX, Raw data exported from the Synapt G2S Model Mass Spectrometer (WATERS), applied for data analyses and preparation for the sequence of spot D in table 1

- D_FUNC001.IDX, Raw data exported from the Synapt G2S Model Mass Spectrometer (WATERS), applied for data analyses and preparation for the sequence of spot D in table 1

- D_FUNC003.STS, Raw data exported from the Synapt G2S Model Mass Spectrometer (WATERS), applied for data analyses and preparation for the sequence of spot D in table 1

- D_HEADER.TXT, Raw data exported from the Synapt G2S Model Mass Spectrometer (WATERS), applied for data analyses and preparation for the sequence of spot D in table 1
